# Vertebral vascular canal dysplasia in cats: signalment, CT and MRI characteristics, and prevalence

**DOI:** 10.3389/fvets.2025.1642066

**Published:** 2025-08-01

**Authors:** Q. Van Koulil, K. M. Santifort, D. S. Willems, G. Bolen, S. De Decker, M. Bernardini, N. Bergknut, I. Van Soens

**Affiliations:** ^1^IVC Evidensia Small Animal Referral Hospital “Hart van Brabant” Neurology, Waalwijk, Netherlands; ^2^IVC Evidensia Small Animal Referral Hospital Arnhem Neurology, Arnhem, Netherlands; ^3^Teaching and Clinical Department of Companion Animal, Faculty of Veterinary Medicine, Fundamental and Applied Research for Animals & Health (FARAH), University of Liège, Liège, Belgium; ^4^Department of Clinical Science and Services, The Royal Veterinary College, Hatfield, United Kingdom; ^5^AniCura Portoni Rossi Veterinary Hospital, Bologna, Italy; ^6^Department of Animal Medicine, Productions and Health, University of Padua, Padua, Italy; ^7^Department of Small Animal Clinical Sciences, Faculty of Veterinary Medicine, University of Liège, Liège, Belgium

**Keywords:** feline, vertebral, vascular canal, dysplasia, VVCD, congenital, malformation

## Abstract

**Introduction:**

Numerous vertebral anomalies have been characterised in dogs, whereas congenital vertebral malformations in cats have been less frequently described. The aim of the present study was to report and describe a vertebral malformation in cats recently reported in dogs—vertebral vascular canal dysplasia (VVCD)—to apply the previously reported scoring system to a feline study population, and to evaluate its inter- and intra-observer agreement.

**Material and methods:**

CT and MRI studies from five different feline populations were retrospectively evaluated. Patients were included as VVCD-affected if they showed single or multiple VVCDs of the thoracic vertebral column and were scored using the previously published canine scoring system.

**Results:**

A total of 2,037 cats were evaluated, of which 541 (26.6%) were found to have VVCD. In addition to the thoracic vertebrae, cervical and lumbar vertebrae were also affected. Different score distributions were observed across thoracic vertebrae, suggesting a possible regional pattern. Most patients underwent CT (508), while only 33 had MRI.

**Discussion:**

CT was considered superior due to its higher spatial resolution and the availability of axial/transverse images for all vertebral bodies, which allowed improved visualisation of vertebral body anatomy and better evaluation of the form, shape, and depth of the dysplastic vascular canals. Especially in more subtle cases, transverse views were necessary to confirm the presence and assess the extent of VVCD. Intra- and interobserver agreement was variable (range 0.543-1.000 and 0.225-0.894, respectively) depending on adjustments to the scoring system, reflecting the role of subjectivity in the interpretation of VVCD with this system. Clinical relevance was not assessed. Future studies are required to investigate prevalence, explore possible etiologies, and determine the potential clinical significance of VVCD in feline spinal disease.

## 1 Introduction

Numerous vertebral anomalies in dogs have been described in previously published studies (1). Brachycephalic dog breeds like pugs and “screw-tailed” dog breeds such as French and English Bulldogs have been overrepresented (2). Individual dogs can often have multiple vertebral anomalies leading to vertebral misalignment, kyphosis and scoliosis of the vertebral column. Clinical relevance of these anomalies seems variable, as not all dogs displaying these abnormalities show compatible clinical signs (3). Nevertheless, these anomalies may play a role in treatment of other spinal cord disease, potentially impacting surgical planning and increasing the risk of complications.

Santifort et al. (4) published a study of a vertebral anomaly not previously reported in dogs (2), which the authors named “vertebral vascular canal dysplasia” (VVCD). The anomaly was defined as *“a defect in the ossification of the vertebral body of* var*iable extent, centred around the position of the vascular canal*” (4). This study aimed to introduce a description and scoring system of this VVCD that could be used as background for future studies and to report the prevalence in French and English bulldogs.

Congenital vertebral anomalies, like hemi-, block vertebrae (5, 6), spina bifida (7), meningo(myelo)cele (8) and thoracic vertebral stenosis (9) have been described in feline patients.

To the author’s knowledge VVCD, as published in dogs, has not been described in cats.

This multicentric, retrospective, observational study aimed to (1) report the prevalence and describe the signalment of cats with VVCD affecting the thoracic vertebral column in this population, (2) describe CT and MRI characteristics of VVCD in cats, and (3) report the intra- and interobserver agreement when scoring VVCD in cats.

We hypothesise that (1) this vertebral body malformation is often seen as an incidental finding when imaging studies of the thorax or thoracic vertebral column are performed as a diagnostic tool for different clinical pathologies, and (2) this disorder is less commonly seen in cats than in the reported brachycephalic dog breeds as evaluated by CT [reported prevalence of >68% (4)].

## 2 Materials and methods

### 2.1 Study population and study design

This multicentric, retrospective, observational study included feline patients presented between 2018 and 2023 at IVC Evidensia Small Animal Referral Hospital “Hart van Brabant,” Waalwijk (The Netherlands), Teaching and Clinical Department of Companion Animal, Faculty of Veterinary Medicine, University of Liege (Belgium), The Royal Veterinary College, (United Kingdom), Anicura Veterinary Hospital “Portoni Rossi,” Bologna (Italy). Clinical information and diagnostic imaging were retrieved retrospectively.

Inclusion criteria for each feline patient required availability of the following data for analysis: (1) a CT or MRI study of the entire thoracic vertebral column; (2) to be included as VVCD-affected, at least one vertebra had to show evidence of VVCD. For patients who underwent an MRI, a sagittal T2W of the entire thoracic vertebral column had to be available, even if transverse sequences were not available at every vertebra, they were included when a VVCD was suspected based on changes seen on the sagittal view; (3) clinical records, including data on breed and sex. Age at the time of diagnostic imaging was recorded if available, but it was not a necessary inclusion criterion.

CT and MR images of feline cases with VVCD were collected from the various databases by an ECVN resident (QK). The Dutch and Belgian feline patients included in the study were evaluated by their respective ECVDI diplomate (DW and GB, respectively), and the UK and Italian populations were evaluated by an ECVN resident (QK). MRI studies were performed on a 1.5 T scanner (Vantage Elan, Canon Medical Systems Europe B.V., Netherlands and GE Healthcare, Signa Explorer, Milwaukee, Wisconsin). Details of MRI studies including specific sequences performed and for instance NAQ (number of acquisitions), TR (repetition time), and TE (echo time) were not recorded.

CT studies were performed on an 80-slice scanner (Canon Aquilion, Japan), a 64-slice scanner (Siemens Somatom Confidence, Germany), or a 320-slice helical multidetector CT scanner (Canon Aquilion ONE/GENESIS, Japan). The clinical records did not contain information regarding the indications for performing the MRI and CT examinations.

### 2.2 Definition and scoring criteria for VVCD anomalies

VVCD was defined as “*a defect in the ossification of the vertebral body of* var*iable extent, centred around the position of the vascular canal*” (4). The VVCD-scoring system provided by the canine study of Santifort et al (4). was used to evaluate and score the vertebral canal abnormalities of the feline patients included in this study.

The VVCD in the vertebrae was scored as A = maximum 50% of the vertebral body height (VBH), B = 50- < 100% of VBH, C = complete (100% of VBH). The morphology of the canal is categorized as: s = single (midline or unilateral), d = double (bilateral), or c = complex (double and converging to single or deviating in shape).

**Figure 1:**
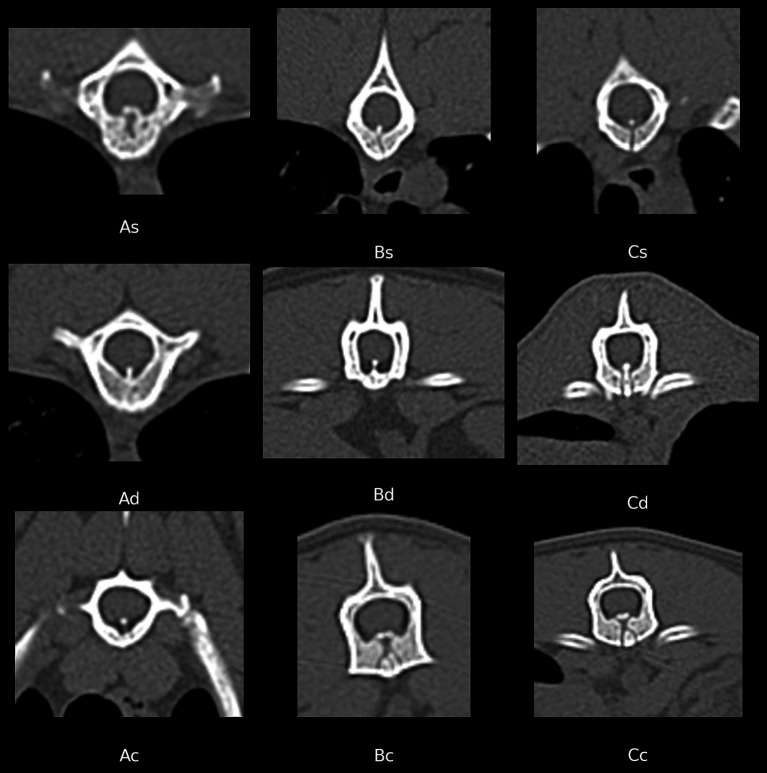
Transverse CT images of different vertebrae (in bone window) showing examples of the different vertebral vascular canal dysplasia (VVCD) grades, based on the scoring system of Santifort et al. 2022. The first letter defined the height of the affected vertebral body. Score A = max. 50% of the vertebral body height (VBH), B = 50 - < 100% of VBH, C = complete (100% of VBH). The second letter described the shape of canal: s = single (midline or unilateral), d = double (bilateral), or c = complex (double and converging to single or deviating in shape).

Transverse CT images of selected examples of the different forms of VVCD in cats of our study population are included in Figure 1.

#### 2.2.1 Vertebral vascular canal dysplasia scores

VVCD was scored based on the criteria set by Santifort et al. (4), where vertebrae T1 through T13 were evaluated. If there was an “extra” thoracic vertebra with either uni- or bilateral ribs, this was seen as a 14th thoracic vertebra and scored. If cervical or lumbar vertebrae had a similar dysplasia seen on the provided DICOM files, these were also scored and recorded. If any other vertebral anomalies were seen in any vertebra, this was recorded.

**Figure 2:**
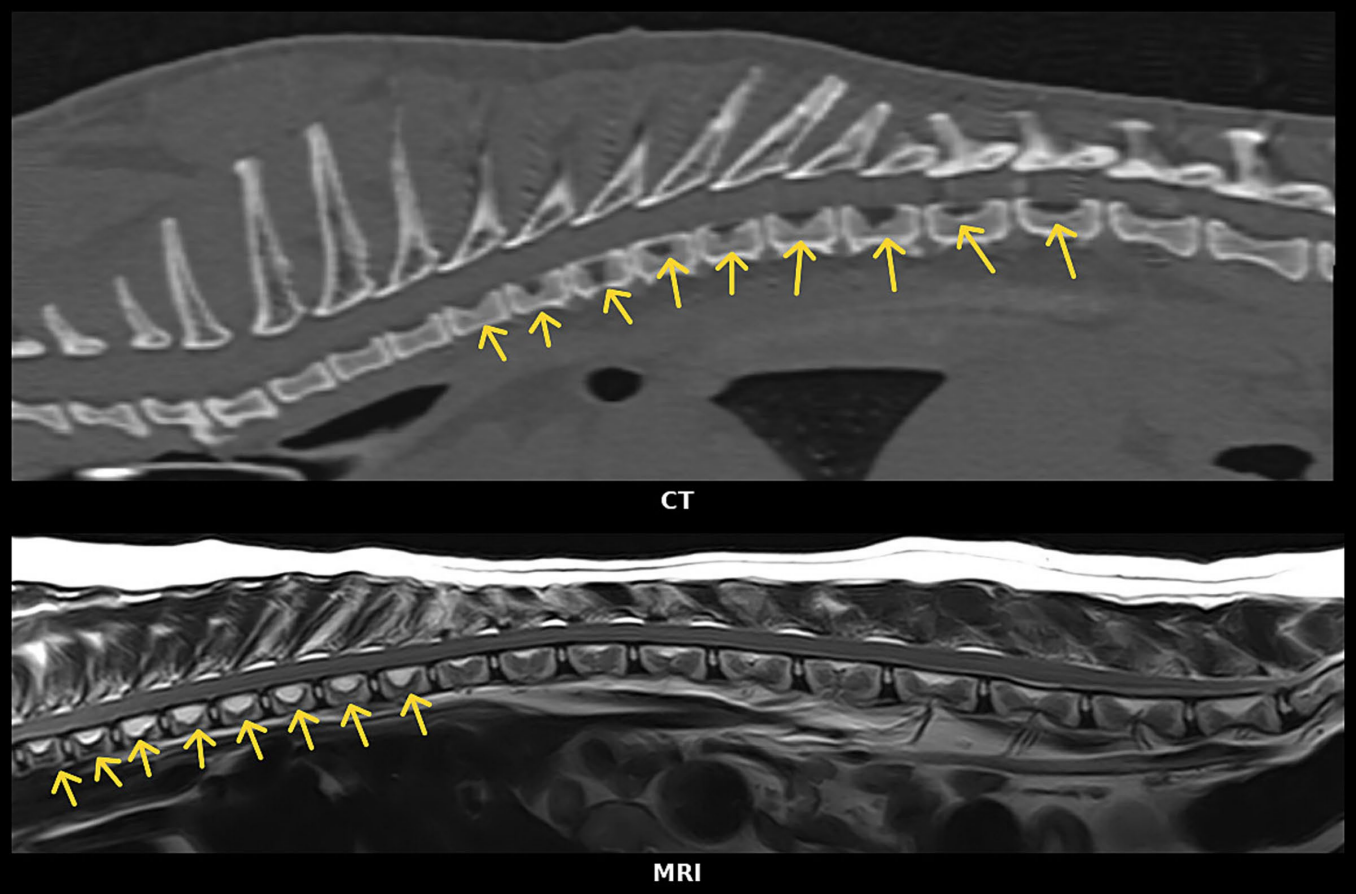
(Top) Sagittal CT reconstruction image of the thoracic vertebral column (bone window). (Bottom) Sagittal T2W MR image of the thoraco-lumbar vertebral column both showing changes in different vertebrae suspected of vertebral vascular canal dysplasia (VVCD). Yellow arrows indicate thoracic vertebrae suspected of having VVCD.

Reconstruction of image planes for evaluation of VVCD on CT images was allowed (e.g., transverse image planes as well as dorsal and sagittal). Sagittal CT and MRI image examples are included in Figure 2. For the CT studies, a bone window was applied to evaluate the vertebrae for VVCD, and window width and level were adjusted manually where deemed appropriate by the observers [window width (WW): 2,000–3,000 HU; window level (WL): 300–500 HU]. If the bone window was not available, the evaluation was performed using a soft tissue window (WW: 100–400 HU; WL: around 50 HU).

For the MRI studies, the vertebrae were evaluated based on sagittal and, if available, transverse views. Not all vertebrae were evaluated by transverse sequences, making it impossible to thoroughly evaluate all affected vertebrae on MRI studies. In cases where this evaluation could not be performed or if it was unclear whether there was a VVCD present, the vertebrae were not scored. When a patient had both CT and MRI imaging studies, the CT images were used to evaluate the patient for VVCD.

### 2.3 Intra- and interobserver agreement

To evaluate agreement among independent observers and analyze intraobserver agreement, a subset of randomized VVCD cases was included in a test protocol. This test protocol consisted of both sagittal and transverse still images from DICOM studies of 50 cats with VVCD from all included geographical areas.

The test protocol comprised two sequential steps:

1. Evaluation of sagittal CT and MRI images to determine patient eligibility, based on the presence of at least one vertebral vascular canal dysplasia (VVCD); no transverse images were assessed at this stage.
2. Evaluation of transverse CT and MRI images of the affected vertebrae to determine a VVCD score, using the previously described scoring system; sagittal images were not reassessed in this step.

Each reviewer independently assessed the imaging studies on two separate occasions, with a two-week interval between assessments.

To evaluate intra- and inter-observer agreement, Cohen’s simple unweighted kappa was used. Agreement was calculated under four different analytical scenarios, each reflecting a different level of scoring complexity:

- *Scenario 1—ABC + morphology*: Full scoring system using both vertebral body height classification (A, B, or C) and canal shape (s = single, d = double, c = complex).
- *Scenario 2—ABC (VBH only, no morphology)*: Simplified analysis including only vertebral body height scores, excluding the morphological canal classification.
- *Scenario 3—BC only + morphology*: Includes only B and C scores (>50% VBH involvement) with morphological categories.
- *Scenario 4—BC only (VBH only, no morphology)*: Simplified analysis evaluating only B and C scores, excluding canal morphology.

These scenarios were designed to investigate the effects of different score components—specifically morphology and VBH thresholds—on observer agreement.

Interpretation of Cohen’s kappa scores followed standard thresholds: poor agreement (<0), slight agreement (0.00–0.20), fair agreement (0.21–0.40), moderate agreement (0.41–0.60), substantial agreement (0.61–0.80), and almost perfect agreement (0.81–1.00; 1.00 = perfect agreement).

## 3 Results

### 3.1 Signalment of the population of cats

#### 3.1.1 Breed distribution

**Figure 3:**
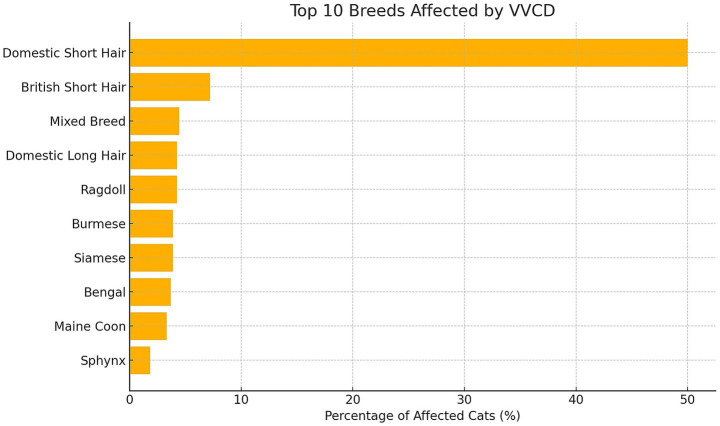
Overview of the top 10 cat breeds with vertebral vascular canal dysplasia (VVCD) and their breed prevalence in percentages relative to the total number of affected cats that were evaluated.

A total of 2,037 cats were evaluated; 541 (26.6%) were found to have VVCD and met the inclusion criteria. Domestic Short Hair was the most commonly affected breed (271/541, 50.0%). Other breeds included: British Short Hair (39/541, 7.2%), Mixed Breed (24/541, 4.4%), Ragdoll (23/541, 4.3%), Siamese (21/541, 3.9%), Burmese (21/541, 3.9%), Domestic Long Hair (23/541, 4.3%), Bengal (20/541, 3.7%), Maine Coon (18/541, 3.3%), Sphynx (10/541, 1.9%), British Blue (7/541, 1.3%), six (1.1%) of the following breeds; Birman, Moggie, Siberian, Persian, British Long Hair. Three (0.6%) of the following breeds: Tonkinese, Exotic Shorthair, Russian Blue, Egyptian Mau, Abyssinian. Two (0.4%) of the following breeds: Domestic Medium Hair, Bombay, Balinese, Cornish Rex, Norwegian Forest, Oriental Short Hair. One (0.2%) of the following breeds: Savannah, Ocicat, Devon Rex, Selkirk Rex, Turkish Angora, Korat and Turkish Van. Figure 3 shows the distribution of the top 10 affected breeds by VVCD.

#### 3.1.2 Gender

With respect to gender distribution, male cats were seen in 312 cases (57.7%), while females accounted for 229 cases (42.3%).

#### 3.1.3 Age at the time of diagnostic imaging

Age at the time of diagnostic imaging was available for 529 out of 541 cats (97.8%). Ages ranged from 2 to 252 months. The mean age was 90.1 months (±46.3 SD), and the median was 96 months. Age was not available for 12 cats (2.2%).

### 3.2 Modality

Most of the cats had a CT scan performed (508/541, 93.9%) compared to an MRI (33/541, 6.1%).

### 3.3 Evaluated vertebrae and distribution of vertebral vascular canal dysplasia scores

#### 3.3.1 Detailed description of distribution of VVCD scores per thoracic vertebra

**Figure 4:**
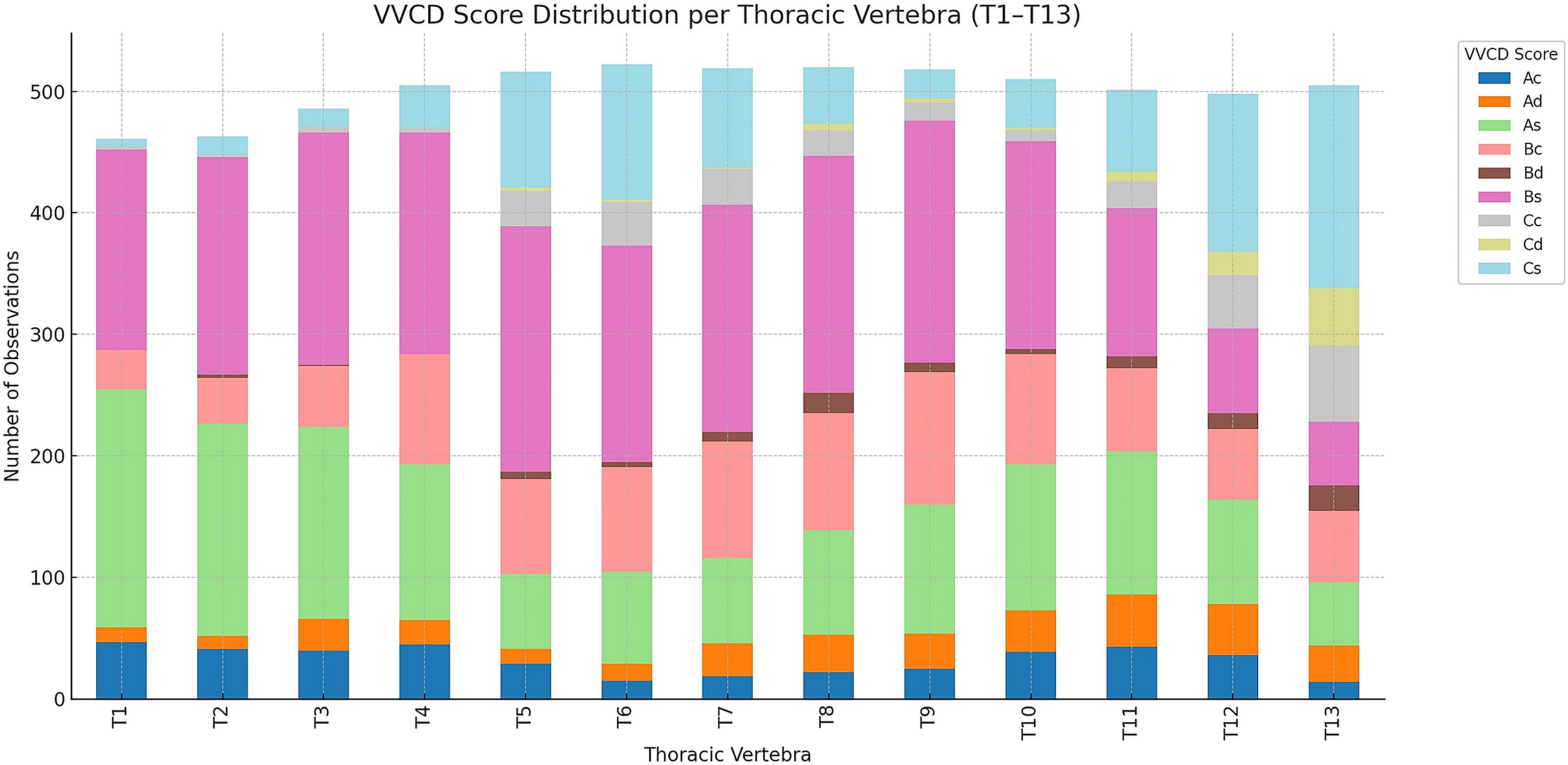
Overview of the distribution of vertebral vascular canal dysplasia (VVCD) per thoracic vertebrae evaluated. Thoracic vertebrae are seen on the horizontal axis, and numbers compared to the total number of vertebrae are on the vertical axis. The different VVCD grades are shown in different colors corresponding to the legend on the right.

Across thoracic vertebrae T1 to T13, distinct patterns emerged in the distribution of VVCDs, shown in Figure 4.

At the cranial thoracic level, T1 was predominantly scored as As (190/439; 43.3%) and Bs (130/439; 29.6%), with lesser occurrences of Bc (45/439; 10.3%), Cs (37/439; 8.4%), and Ac (18/439; 4.1%). Rarely encountered classifications included Ad (15/439; 3.4%) and Cd (4/439; 0.9%). T2 showed a similar distribution, with As (195/465; 41.9%) being most frequent, followed by Bs (123/465; 26.5%), Cs (57/465; 12.3%), Bc (47/465; 10.1%), and Ad (23/465; 4.9%). Ac and Cd were also observed at 14/465 (3.0%) and 6/465 (1.3%), respectively. T3 continued this pattern, with As (155/487; 31.8%) and Bs (137/487; 28.1%) as the leading scores, accompanied by Bc (90/487; 18.5%), Cs (38/487; 7.8%), and Ad (33/487; 6.8%). Ac and Cd were present at 31/487 (6.4%) and 3/487 (0.6%), respectively.

From T4 to T9 (mid-thoracic region), Bs remained the dominant score across most levels. T4 exhibited Bs in 160/499 cases (32.1%), followed by Bc (136/499; 27.3%), As (79/499; 15.8%), and Cs (60/499; 12.0%). Additional scores included Ac (24/499; 4.8%), Ad (17/499; 3.4%), and Cc (23/499; 4.6%). T5 showed the highest count of Bs (187/498; 37.6%), along with significant scores of Cs (92/498; 18.5%), As (102/498; 20.5%), and Bc (78/498; 15.7%). Other scores like Ad (29/498; 5.8%) and Ac (10/498; 2.0%) were also present. T6 was marked by the peak presence of Bs (222/519; 42.8%), with notable counts of As (139/519; 26.8%), Cs (89/519; 17.1%), and Bc (46/519; 8.9%). Ac and Ad occurred in 7/519 (1.3%) and 16/519 (3.1%) cases, respectively.

T7 and T8 continued this trend, each with Bs accounting for over 50% of scored vertebrae (T7: 257/500; 51.4%, T8: 254/507; 50.1%). As (T7: 97/500; 19.4%, T8: 108/507; 21.3%), Cs (T7: 92/500; 18.4%, T8: 98/507; 19.3%), and Bc (T7: 54/500; 10.8%, T8: 47/507; 9.3%) also appeared prominently. At T9, Bs again led (230/518; 44.4%), with substantial presence of Cs (96/518; 18.5%), As (104/518; 20.1%), and Bc (62/518; 12.0%). Ad and Ac were observed at 13/518 (2.5%) each.

In the caudal thoracic region, from T10 to T13, there was a notable shift toward increased proportions of Cs, Cc, and Cd. T10 displayed Bs as the most common score (209/518; 40.3%), followed by Cs (109/518; 21.0%), Bc (88/518; 17.0%), and As (77/518; 14.9%). Minor score categories included Ac (14/518; 2.7%), Ad (15/518; 2.9%), and Cd (6/518; 1.2%). T11 presented a more balanced distribution, with Bs (141/363; 38.8%) and As (99/363; 27.3%), along with Cs (59/363; 16.3%), Cc (31/363; 8.5%), and Ad (33/363; 9.1%). At T12, Cs and Ac became the most frequent scores (108/471; 22.9% each), with strong representation of As (86/471; 18.3%), Bc (81/471; 17.2%), and Cc (52/471; 11.0%). Ad occurred in 36/471 (7.6%) of cases. T13 showed a sharp increase in Cs (132/575; 23.0%) and Bc (136/575; 23.7%), followed by Cc (77/575; 13.4%), Ac (132/575; 23.0%), and Ad (92/575; 16.0%). As and Bs dropped substantially (2/575; 0.3% and 3/575; 0.5%, respectively), while Cd remained low (1/575; 0.2%).

Overall, Bs was the most consistently observed score across all thoracic levels, particularly in the mid-thoracic region (T4–T9). Cs, Cc, and Cd scores increased markedly toward the caudal region, suggesting a possible regional pattern in VVCD expression. As, Bc, and Ad occurred more frequently in the cranial and mid-thoracic vertebrae, indicating potential regional specificity of these grades of VVCD.

**Figure 5:**
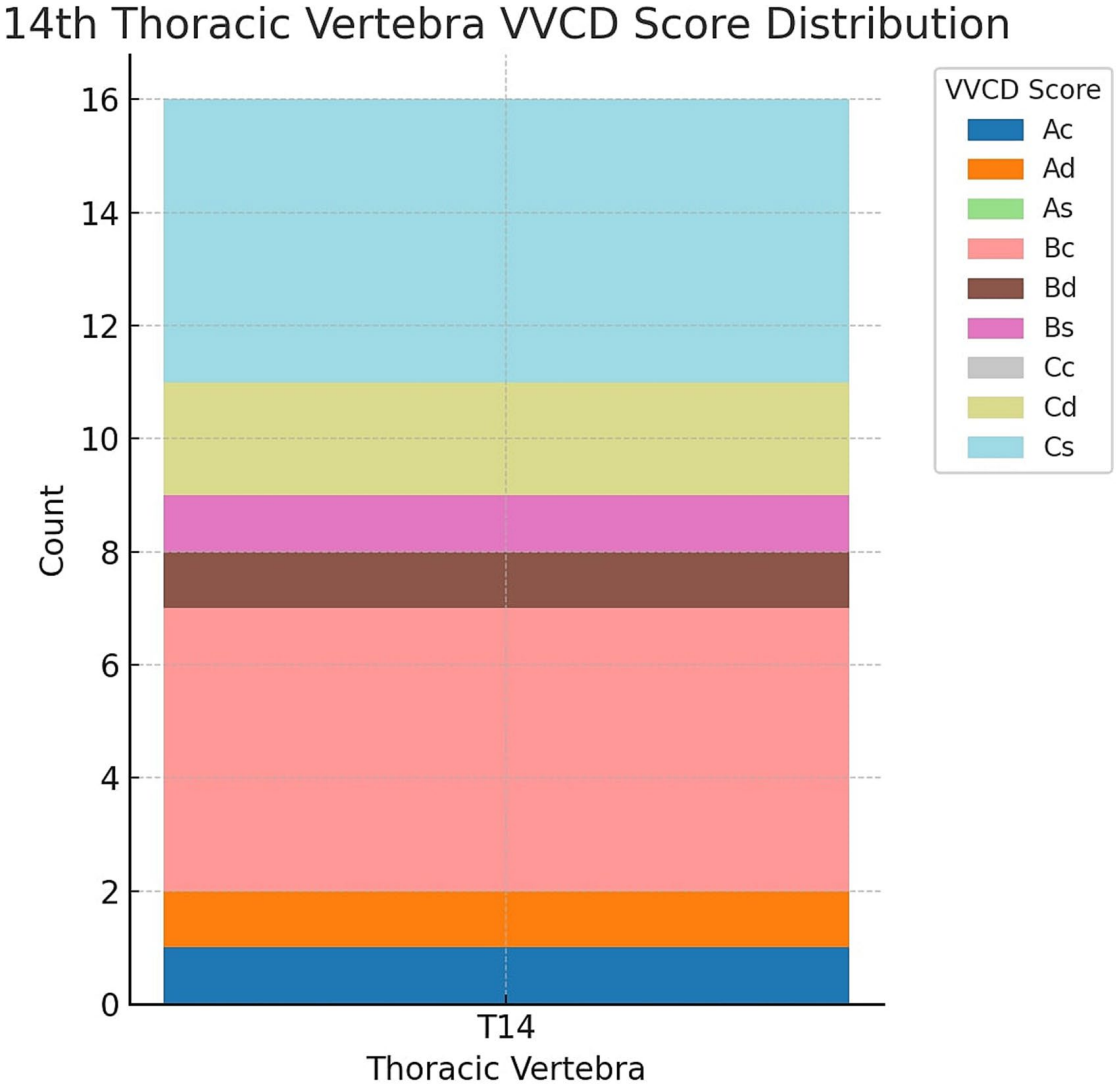
Distribution of vertebral vascular canal dysplasia (VVCD) scores seen at the 14th thoracic vertebrae. Thoracic vertebrae are seen on the horizontal axis, and the number of affected vertebrae are seen on the vertical axis. The different VVCD grades are shown in different colors corresponding to the legend on the right.

During the imaging studies’ evaluation, a 14th thoracic vertebra was identified in 16 out of 541 cats (2.96%). Figure 5 illustrates the different grades of these “extra” thoracic vertebrae. The most common VVCD scores in these vertebrae were Bc (5/16; 31.2%) and Cs (5/16; 31.2%), followed by Cd (2/16; 12.5%), Ad (1/16; 6.2%), Bd (1/16; 6.2%), Ac (1/16; 6.2%), and Bs (1/16; 6.2%).

#### 3.3.2 Cervical and lumbar VVCD

**Figure 6:**
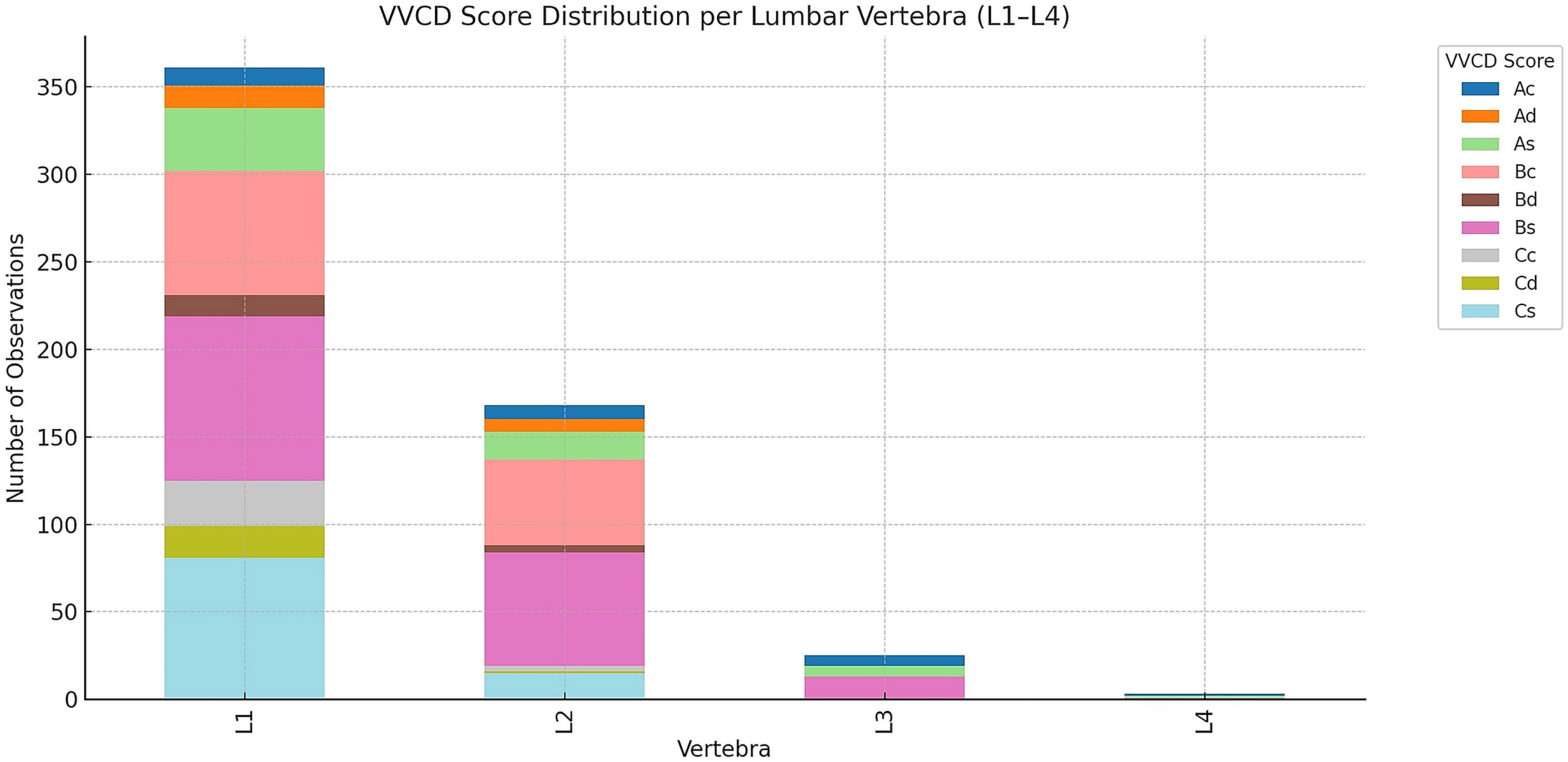
Overview of the distribution of vertebral vascular canal dysplasia (VVCD) per lumbar vertebrae evaluated. Affected lumbar vertebrae are seen on the horizontal axis, and numbers compared to the total number of vertebrae are on the vertical axis. The different VVCD grades are shown in different colors corresponding to the legend on the right.

Besides the thoracic vertebrae, a total of 557 lumbar vertebrae were found to be affected. L1 was the most frequently affected (361/557; 64.8%), followed by L2 (168/557; 30.2%), L3 (25/557; 4.5%), and L4 (3/557; 0.5%). Of all imaging studies where lumbar vertebrae were visible and identified as having VVCD, only the first four lumbar vertebrae were consistently visible. Figure 6 illustrates the different grades per lumbar vertebra.

In L1, the most common score was Bs (94/361; 26.0%), followed by Cs (81/361; 22.4%), Bc (71/361; 19.7%), As (36/361; 10.0%), Cc (26/361; 7.2%), Cd (18/361; 5.0%), Ad (13/361; 3.6%), Bd (12/361; 3.3%), and Ac (10/361; 2.8%).

In L2, scores included Bs (65/168; 38.7%), Bc (49/168; 29.2%), As (16/168; 9.5%), Cs (15/168; 8.9%), Ac (8/168; 4.8%), Ad (7/168; 4.2%), Bd (4/168; 2.4%), Cc (3/168; 1.8%), and Cd (1/168; 0.6%).

In L3, the most common score was Bs (13/25; 52.0%), followed by As (6/25; 24.0%) and Ac (6/25; 24.0%).

In L4, the scores were evenly distributed: Ac (1/3; 33.3%), As (1/3; 33.3%), and Bs (1/3; 33.3%).

In three patients evaluated, changes were also seen in the cervical vertebrae. The following abnormalities were noticed. In one cat, the C7 vertebrae showed a Bs score; in one cat, C2 until C7 showed VVCD, which was scored as As and in the last cat, the C3 until the C7 vertebrae showed a VVCD scored as Ac.

Besides the VVCD abnormalities visible in the thoracic, lumbar, and cervical vertebrae, no other vertebral abnormalities were seen on the evaluation of these patients.

### 3.4 Intra- and interobserver agreement

#### 3.4.1 Intra-observer agreement

Intra-observer agreement was assessed across all four scoring scenarios. The observed kappa values ranged from 0.543 to 1.000, reflecting moderate to perfect agreement depending on the scenario and observer.

These results are summarized in Table 1, for the detailed values for each observer and per scenario the reader is referred to the Supplementary file 1.

**Table 1:** Table containing the ranges of calculated simple unweighted Cohen’s kappa scores between the different scenarios for the intra-observer agreement and of both evaluations of the inter-observer agreement.

Scenario	Intra-observer (*κ*)	Inter-observer 1st (*κ*)	Inter-observer 2nd (*κ*)
Scenario 1—ABC + morphology	0.543–0.696	0.246–0.419	0.244–0.621
Scenario 2—VBH only (ABC)	0.584–0.773	0.346–0.647	0.462–0.644
Scenario 3—BC only + morphology	0.668–0.849	0.401–0.543	0.225–0.686
Scenario 4—BC only (no morphology)	0.622–1.000	0.588–0.894	0.777–0.790

Scenario 1 (ABC + morphology), the most elaborate and complicated scoring scheme, yielded the lowest overall intra-observer agreement, with values ranging from 0.543 to 0.696. Scenario 2 (ABC, VBH only) demonstrated improved agreement (*κ* = 0.584 to 0.773), suggesting that excluding canal morphology contributed to more consistent scoring. Scenario 3 (BC only + morphology) further improved reliability (*κ* = 0.668 to 0.849) by focusing on vertebrae with greater VBH involvement. The highest intra-observer agreement was observed in Scenario 4 (BC only, no morphology), with kappa values ranging from 0.622 to 1.000, including one observer achieving perfect agreement.

#### 3.4.2 Inter-observer agreement

Inter-observer agreement was evaluated separately for the first and second assessment rounds. In the first evaluation, kappa values ranged from 0.246 to 0.894 across all observer pairs and scenarios. In the second evaluation, inter-observer agreement ranged from 0.225 to 0.790.

These results are summarized in Table 1, for the detailed values for each observer and per scenario the reader is referred to the Supplementary file 1.

Scenario 1 showed the lowest inter-observer consistency, with kappa values ranging from 0.246 to 0.419 in the first evaluation and 0.244 to 0.621 in the second. Scenario 2 showed improved inter-observer agreement (*κ* = 0.346 to 0.647 in the first and 0.462 to 0.644 in the second), suggesting that removing morphology enhanced agreement. Scenario 3 yielded fair to substantial inter-observer agreement, with more variation between rounds (*κ* = 0.401 to 0.543 in the first and 0.225 to 0.686 in the second). The highest and most consistent inter-observer agreement was observed in Scenario 4, with values ranging from 0.588 to 0.894 in the first evaluation and 0.777 to 0.790 in the second.

## 4 Discussion

### 4.1 VVCD in feline patients

This study represents the first large-scale investigation of vertebral vascular canal dysplasia (VVCD) in a feline study population, implementing the scoring methodology introduced by Santifort et al. (4). Out of 2,037 cats assessed through imaging studies, 541 (prevalence of 26.6%) demonstrated evidence of VVCD in at least one thoracic vertebra. Although this prevalence suggests that VVCD is fairly common in cats, the prevalence is much lower than that in French and English bulldogs as evaluated by CT in a UK population (>68%) (4). The most commonly affected breed was the Domestic Shorthair (DSH), with a mild overrepresentation of male cats. This seems to align with the demographic distribution of the general population of cats presenting for diagnostic imaging, suggesting that VVCD may not be breed-specific and may occur sporadically across a wide genetic pool.

The distribution of dysplastic changes was variable, with the thoracic vertebrae most commonly affected. The sixth thoracic vertebra (T6) was the most frequently involved vertebra. Although not the primary focus of the study, lesions were also identified in the lumbar and, less frequently, in the cervical vertebrae. As this was not the primary focus of the study, the number of affected vertebrae in other regions may be underestimated. This does support the notion that VVCD is not restricted to the thoracic spine and that comprehensive evaluation of the entire vertebral column should be considered when this anomaly is suspected. Notably, affected vertebrae displayed other congenital anomalies in some dogs in a previous study, a feature not recognised in this study (1, 4).

In this study, only a small proportion of cats (2.96%) were found to have a “14th” thoracic vertebra. This is notably lower than the 14% prevalence of a “thoracicized” L1 reported in a previous study evaluating transitional vertebrae in feline patients (6). As the lumbar vertebrae were not the focus of the present study, this number may nevertheless represent an underestimation.

### 4.2 Clinical relevance

While it was not the aim of this study to evaluate the clinical significance of VVCD in cats, we shortly reflect on this matter here. The clinical relevance of VVCD in cats remains uncertain. In our study, 508 cats underwent CT evaluation, likely reflecting a high number of referrals for thoracic or pulmonary conditions, as well as emergency presentations such as trauma. However, because the specific clinical indications for imaging were not systematically retrieved from the medical records, we cannot draw definitive conclusions.

From the 541 cats evaluated only 54 were presented for neurological evaluation. As detailed neurological examinations were not systematically recorded or retrieved from clinical records, we cannot say if these patients were presented for, for instance, paraparesis/-plegia and ataxia or other neurological abnormalities and if the VVCD played a role therein or not. In canine literature, VVCD has been speculated to play a role in spinal cord compression or impaired venous drainage (1), but no such relationship has been established, let alone in cats. Thus, VVCD should currently be regarded as a potentially incidental finding with unclear clinical consequences. Chronic progressive thoracolumbar myelopathy, like vertebral canal stenosis (9), has been reported in cats, although not very often. It is still unclear whether VVCD also leads to similar long-term neurological problems. If it does, this condition might become relevant during spinal surgeries. Although rare in practice, VVCD could affect spinal stabilisation in cats with spinal trauma, because the affected vertebrae might not be strong enough to hold surgical implants securely. To better understand this, prospective studies with standardised neurological examinations are needed.

### 4.3 Inter- and intra-observer agreement of the scoring system

The scoring system applied in this study was adapted from a canine model (4) and integrates assessments of vertebral body height (VBH) and vascular canal morphology. While this semi-quantitative framework provided a structured approach to evaluating vertebral vascular canal dysplasia (VVCD), its application in cats revealed interpretative challenges. Disagreements were particularly evident in borderline cases, where observers differed in determining whether VBH involvement was below or above the 50% threshold and in classifying the canal shape as single, double, or complex. These discrepancies indicate the presence of a grey zone between normal anatomical variation and dysplastic changes. Based on observed scoring variability, we propose that vertebrae with >50% VBH involvement (scores B or C), accompanied by clearly abnormal canal morphology, represent the most definitive examples of VVCD. In contrast, the distinction between score A and truly “normal” vertebrae remains ambiguous. Establishing a reliable threshold for “normal” versus “abnormal” will require morphometric studies in clinically healthy feline populations. Recent feline imaging research describing the dimensions of the vertebral canal (10) and intervertebral discs (11) offers a foundation for such datasets, which may help define anatomical boundaries and reduce subjective interpretation.

Despite the use of this scoring system, observer variability remained a significant factor. Intra-observer agreement ranged from moderate to perfect (*κ* = 0.543 to 1.000), indicating that individual observers were generally consistent with themselves across time, though this varied by scenario and complexity. Inter-observer agreement was more variable and generally lower, ranging from fair to almost perfect in the first evaluation round (*κ* = 0.246 to 0.894), and from fair to substantial in the second round (*κ* = 0.225 to 0.790). The lowest levels of agreement were observed in Scenario 1, which included both VBH and canal morphology classification, reflecting the subjectivity introduced by morphological interpretation. In contrast, Scenario 4, which focused only on vertebrae with >50% VBH involvement (scores B and C) and excluded morphology scoring, resulted in the highest and most consistent agreement between and within observers. Observer II consistently demonstrated lower inter-observer agreement across scenarios, suggesting that factors such as clinical experience, interpretive thresholds, or familiarity with the scoring criteria may significantly affect reproducibility.

The authors highlight the challenges associated with applying the current semi-quantitative scoring system, which was adapted from a canine model and may not be directly suitable for use in feline patients. While this system provides a useful starting point, it requires refinement to address species-specific anatomical differences. Enhancing the scoring model—alongside the development of standardized reference data and targeted observer training—will likely improve diagnostic reliability and consistency. In turn, this will support more accurate evaluation of this type of vertebral abnormalities, help define the boundary of what is consider “normal” and “abnormal,” and hopefully clarify the clinical relevance of vertebral vascular canal dysplasia (VVCD) in future studies. In any case, the application of the scoring system may help to (semi-)quantify VVCD, enabling categorization and facilitate distinguishment of “normal” and “abnormal” in future studies.

### 4.4 Pathogenesis

The pathogenesis of VVCD in cats remains speculative. Santifort et al. (4) postulated that dysplastic vascular canals may arise from aberrant formation or remodelling of the basivertebral veins during embryogenesis. Normally, venous drainage structures form within the vertebral bodies and are accompanied by corresponding bony canals. Disruption in this process—such as abnormal venous development, excessive pressure, or altered ossification—may result in unusually wide or misshapen canals (12–15). While this theory remains unverified in dogs or cats, abnormal bony vascular channels on CT have been documented in canine lumbar vertebrae (16). However, no detailed anatomical description exists confirming the presence or morphology of such vertebral canals in cats.

Moreover, the anatomy and development of the basivertebral venous canal and vertebral venous plexus—well described in humans (17)—have not been adequately studied in felines, compared to canine patients (18–20). The absence of data on these structures limits our ability to determine whether VVCD represents a malformation, an anatomical variant, or a species-specific adaptation. Significantly, VVCDs in this study were not associated with vertebral anomalies in brachycephalic or dysmorphic cat breeds, further distancing their etiology from the dysregulated vertebral development commonly observed in canine populations.

### 4.5 Limitations

This study has several important limitations, most of which stem from its retrospective design. Also, due to the study’s focus on the thoracic vertebral column, the apparent predominance of the anomaly in this region may reflect sampling bias rather than a true anatomical distribution.

Imaging data were collected from multiple institutions using a variety of CT and MRI scanners, protocols, and patient positioning techniques. None of these imaging procedures were standardized to evaluate vertebral vascular canal dysplasia. As a result, image quality varied considerably across the dataset, including inconsistencies in slice thickness, reconstruction parameters, and anatomical coverage. These technical differences likely influenced the detectability and characterization of these VVCD.

The majority of patients were evaluated using CT, which typically provided superior visualization of vertebral bone structures—particularly with its better spatial resolution and when thin slices are available. However, specific algorithms that would have been optimal for the evaluation of the vertebrae (i.e., bone algorithms) were not available for all CT studies, and as soft tissue windowing has lower window levels and narrower window widths, this may have affected the observers’ ability to assess bony structures like VVCD. For those cases where only an MRI had been performed, the observers’ ability to evaluate VVCD configuration and vertebral body anatomy in three dimensions was hindered for several reasons, including: the lack of transverse planes at the level of all thoracic vertebral bodies; the lack of specific sequences other than only T2W sagittal images; and the differences in scanning parameters per study. For the latter, as the sequence parameters were not standardized, observers may not have been able to evaluate VVCD optimally even when multiple sequences and planes had been acquired. Consequently, some VVCDs may have been underreported or misclassified.

In addition to technical factors, a key limitation of this study lies in the semi-quantitative nature of the scoring system, which—despite being structured—relies heavily on subjective interpretation. Inter-observer agreement varied widely, particularly in scenarios involving both vertebral body height (VBH) and canal morphology, suggesting that distinguishing between borderline categories (e.g., <50% vs. >50% VBH involvement or canal classifications) remains inconsistent. This variability was compounded by the absence of established reference standards for normal feline vertebral canal anatomy, making it difficult to confidently define what constitutes a pathological finding. Although intra-observer agreement was generally higher, results still fluctuated across scoring approaches, reflecting the system’s interpretive flexibility.

These limitations highlight the need for objective, species-specific reference datasets, standardized scoring criteria, and consistent imaging protocols. Future studies would benefit from specific observer training and technical standardization to improve reproducibility and reduce subjectivity in VVCD assessments.

## 5 Conclusion

In conclusion, VVCD appears to be a relatively common finding in cats undergoing imaging of the thoracic vertebral column for different clinical conditions, though less common than previously reported in brachycephalic dog breeds as evaluated by CT scans (4). The scoring system applied in this study may benefit from adjustments to improve intra- and interobserver agreement.

Future work should aim to establish anatomical baselines (i.e., document what is to be regarded as “normal”), refine diagnostic criteria, and explore potential clinical correlations, particularly in cats with spinal disease. Prospective, high-quality CT-based studies will be essential to advance our understanding of this vertebral anomaly.

## Data Availability

The original contributions presented in the study are included in the article/Supplementary material, further inquiries can be directed to the corresponding author.

## References

[1] De Decker S Rohdin C Gutierrez-Quintana R. Vertebral and spinal malformations in small brachycephalic dog breeds: current knowledge and remaining questions. Vet J. (2024) 304:106095. doi: 10.1016/j.tvjl.2024.106095, PMID: 38458418

[2] Gutierrez-Quintana R Guevar J Stalin C Faller K Yeamans C Penderis J. A proposed radiographic classification scheme for congenital thoracic vertebral malformations in brachycephalic “screw-tailed” dog breeds. Vet Radiol Ultrasound. (2014) 55:585–91. doi: 10.1111/vru.12172, PMID: 24833506

[3] De Decker S Packer RMA Cappello R Harcourt-Brown TR Rohdin C Gomes SA. Comparison of signalment and computed tomography findings in French bulldogs, pugs, and English bulldogs with and without clinical signs associated with thoracic hemivertebra. J Vet Intern Med. (2019) 33:2151–9. doi: 10.1111/jvim.15556, PMID: 31407402 PMC6766535

[4] Santifort KM Gutierrez-Quintana R Bernardini M Kortz GD Gomes SA Lowrie M. Vertebral vascular canal dysplasia in French and English bulldogs: clinical, CT, and MRI characteristics and prevalence. Vet Radiol Ultrasound. (2022) 63:281–91. doi: 10.1111/vru.13067, PMID: 35199424

[5] Pollard RE Koehne AL Peterson CB Lyons LA. Japanese bobtail: vertebral morphology and genetic characterization of an established cat breed. J Feline Med Surg. (2015) 17:719–26. doi: 10.1177/1098612X14558147, PMID: 25488973 PMC11104057

[6] Newitt A German AJ Barr FJ. Congenital abnormalities of the feline vertebral column. Vet Radiol Ultrasound. (2008) 49:35–41. doi: 10.1111/j.1740-8261.2007.00314.x, PMID: 18251292

[7] Havlicek M Mathis KR Beck JA Allan GS. Surgical management of vertebral malformation in a Manx cat. J Feline Med Surg. (2009) 11:514–7. doi: 10.1016/j.jfms.2008.11.005 19097923 PMC10832836

[8] Clark L Carlisle CH. Spina bifida with syringomyelia and meningocoele in a short-tailed cat. Aust Vet J. (1975) 51:392–4. doi: 10.1111/j.1751-0813.1975.tb15605.x, PMID: 1103814

[9] Gillespie S De Decker S. Thoracic vertebral canal stenosis in cats: clinical features, diagnostic imaging findings, treatment and outcome. J Feline Med Surg. (2020) 22:1191–9. doi: 10.1177/1098612X20920041, PMID: 32436803 PMC10814363

[10] Ahn S Lee S Kang Y Choi J Yoon J. Morphometric evaluation of the thoracic and lumbar vertebral canal and spinal cord using computed tomography in healthy Korean shorthair cats. Anat Histol Embryol. (2024) 53:e13111. doi: 10.1111/ahe.13111, PMID: 39365153

[11] Richter J Mülling CKW Röhrmann N. A morphometric study on the dimensions of the vertebral canal and intervertebral discs from Th1 to S1 in cats and their relevance for spinal diseases. Vet Sci. (2024) 11:429. doi: 10.3390/vetsci11090429, PMID: 39330808 PMC11435567

[12] Bisland SK Wilson B Burch S. Effect of hypoxic stress on vertebral growth plates. Spine J. (2006) 6:131S. doi: 10.1016/j.spinee.2006.06.245

[13] Murakami U Kameyama Y. Vertebral malformation in the mouse foetus caused by maternal hypoxia during early stages of pregnancy. Development. (1963) 11:107–18. doi: 10.1242/dev.11.1.107

[14] Rivard CH. Effects of hypoxia on the embryogenesis of congenital vertebral malformations in the mouse. Clin Orthop Relat Res. (1986) 208:126. doi: 10.1097/00003086-198607000-00026, PMID: 3720114

[15] Sánchez RC Obregón EB Rauco MR. Hypoxia is an etiological factor in vertebral column deformity of salmon (*Salmo salar*). Aquaculture. (2011) 316:13–9. doi: 10.1016/j.aquaculture.2011.03.012

[16] Jones JC Cartee RE Bartels JE. Computed tomographic anatomy of the canine lumbosacral spine. Vet Radiol Ultrasound. (1995) 36:91–9. doi: 10.1111/j.1740-8261.1995.tb00223.x

[17] Tzika M Paraskevas GK Piagkou M Papatolios AK Natsis K. Basivertebral foramina of true vertebrae: morphometry, topography and clinical considerations. Surg Radiol Anat. (2021) 43:889–907. doi: 10.1007/s00276-021-02690-0, PMID: 33598754

[18] Ariete V Barnert N Gómez M Mieres M Pérez B Gutierrez JC. Morphometrical study of the lumbar segment of the internal vertebral venous plexus in dogs: a contrast CT-based study. Animals. (2021) 11:1502. doi: 10.3390/ani11061502, PMID: 34067340 PMC8224572

[19] Santifort KM Glass EN Meij BP Bergknut N Pumarola M Gil VA. Anatomic description of the basivertebral nerve and meningeal branch of the spinal nerve in the dog. Ann Anat. (2023) 245:152000. doi: 10.1016/j.aanat.2022.152000, PMID: 36183940

[20] Gómez M Freeman L Jones J Lanz O Arnold P. Computed tomographic anatomy of the canine cervical vertebral venous system. Vet Radiol Ultrasound. (2004) 45:29–37. doi: 10.1111/j.1740-8261.2004.04005.x, PMID: 15005358

