# Supplementary material for: Vertebral vascular canal dysplasia in cats: signalment, CT and MRI characteristics, and prevalence
Source: Front Vet Sci. 2025 Aug 1;12:1642066. doi: 10.3389/fvets.2025.1642066 (PMC12353702; doi:10.3389/fvets.2025.1642066)
Supplement: Supplementary file 1 [file Supplementary_file_1.docx]

# Supplementary file 1

# Observer Agreement Analysis – Full evaluation of all four scenarios

This document presents a structured comparison of intra- and inter-observer agreement for four distinct evaluation scenarios, based on Cohen’s unweighted kappa scores.

## Evaluation Scenarios

- **Scenario 1 – ABC + morphology**: Full scoring system using both vertebral body height classification (A, B, or C) and canal shape (s = single, d = double, c = complex).
- **Scenario 2 – ABC (VBH only, no morphology)**: Simplified analysis including only vertebral body height scores, excluding the morphological canal classification.
- **Scenario 3 – BC only + morphology**: Includes only B and C scores (>50% VBH involvement) with morphological categories.
- **Scenario 4 – BC only (VBH only, no morphology)**: Simplified analysis evaluating only B and C scores excluding canal morphology.

## Intra-Observer Agreement

### Scenario 1 – ABC + Morphology

| **Observer** | **Kappa Value** | **Interpretation** |
| --- | --- | --- |
| Observer I | 0.543 | Moderate |
| Observer II | 0.696 | Substantial |
| Observer III | 0.681 | Substantial |

### Scenario 2 – ABC (VBH only, no morphology)

| **Observer** | **Kappa Value** | **Interpretation** |
| --- | --- | --- |
| Observer I | 0.584 | Moderate |
| Observer II | 0.728 | Substantial |
| Observer III | 0.773 | Substantial |

### Scenario 3 – BC Only + Morphology

| **Observer** | **Kappa Value** | **Interpretation** |
| --- | --- | --- |
| Observer I | 0.691 | Substantial |
| Observer II | 0.668 | Substantial |
| Observer III | 0.849 | Almost Perfect |

### Scenario 4 – BC only (VBH only, no morphology)

| **Observer** | **Kappa Value** | **Interpretation** |
| --- | --- | --- |
| Observer I | 0.746 | Substantial |
| Observer II | 0.622 | Substantial |
| Observer III | 1.000 | Almost Perfect |

## Inter-Observer Agreement

### Scenario 1 – First Evaluation

| **Observer Pair** | **Kappa Value** | **Interpretation** |
| --- | --- | --- |
| Observer I vs II | 0.246 | Fair |
| Observer I vs III | 0.419 | Moderate |
| Observer II vs III | 0.364 | Fair |

### Scenario 1 – Second Evaluation

| **Observer Pair** | **Kappa Value** | **Interpretation** |
| --- | --- | --- |
| Observer I vs II | 0.326 | Fair |
| Observer I vs III | 0.621 | Substantial |
| Observer II vs III | 0.244 | Fair |

### Scenario 2 – First Evaluation

| **Observer Pair** | **Kappa Value** | **Interpretation** |
| --- | --- | --- |
| Observer I vs II | 0.346 | Fair |
| Observer I vs III | 0.534 | Moderate |
| Observer II vs III | 0.647 | Substantial |

### Scenario 2 – Second Evaluation

| **Observer Pair** | **Kappa Value** | **Interpretation** |
| --- | --- | --- |
| Observer I vs II | 0.531 | Moderate |
| Observer I vs III | 0.644 | Substantial |
| Observer II vs III | 0.462 | Moderate |

### Scenario 3 – First Evaluation

| **Observer Pair** | **Kappa Value** | **Interpretation** |
| --- | --- | --- |
| Observer I vs II | 0.401 | Fair |
| Observer I vs III | 0.543 | Moderate |
| Observer II vs III | 0.407 | Fair |

### Scenario 3 – Second Evaluation

| **Observer Pair** | **Kappa Value** | **Interpretation** |
| --- | --- | --- |
| Observer I vs II | 0.444 | Moderate |
| Observer I vs III | 0.686 | Substantial |
| Observer II vs III | 0.225 | Fair |

### Scenario 4 – First Evaluation

| **Observer Pair** | **Kappa Value** | **Interpretation** |
| --- | --- | --- |
| Observer I vs II | 0.588 | Moderate |
| Observer I vs III | 0.894 | Almost Perfect |
| Observer II vs III | 0.773 | Substantial |

### Scenario 4 – Second Evaluation

| **Observer Pair** | **Kappa Value** | **Interpretation** |
| --- | --- | --- |
| Observer I vs II | 0.777 | Substantial |
| Observer I vs III | 0.790 | Substantial |
| Observer II vs III | 0.785 | Substantial |
